# Chondroblastoma of the cuboid with an associated aneurysmal bone cyst: a case report

**DOI:** 10.1186/1752-1947-1-135

**Published:** 2007-11-14

**Authors:** Yasir Jamal Sepah, Masood Umer, Khurram Minhas, Kamran Hafeez

**Affiliations:** 1 Aga Khan University Medical College, Karachi-74800, Pakistan; 2 Department of Surgery (Orthopedics) Aga Khan University Hospital, Karachi-74800, Pakistan; 3 Department of Pathology Aga Khan University Hospital, Karachi-74800, Pakistan; 4 Department of Orthopedics Aga Khan University Hospital, Karachi-74800, Pakistan

## Abstract

We report the case of a young adult who presented with a painful foot due to chondroblastoma associated with an aneurismal bone cyst.

Chondroblastoma is a rare benign cartilaginous neoplasm that accounts for approximately 1% of all bone tumors and characteristically arises in the epiphysis of a long bone, particularly the humerus, tibia, and femur. Chondroblastoma can affect people of all ages. It is, however, most common in children and young adults between the ages of 10 and 20 years. Association of chondroblastoma with aneurysmal bone cyst is well documented however this association has only once been reported in the cuboid.

Imaging techniques should be supplemented with an open biopsy for the final diagnosis. Management with curettage, use of high speed burr and bone grafting has shown very good outcomes.

## Introduction

Chondroblastoma is a rare benign cartilaginous neoplasm that accounts for approximately 1 % of all bone tumors and characteristically arises in the epiphysis of a long bone, particularly the humerus, tibia, and femur [1]. Occasionally it can follow a more aggressive course invading the joint spaces, adjacent bones and rarely resulting in metastases[2]. In 1931, Codman classified it as a chondromatous variant of giant cell tumors when he described these lesions in the proximal humerus [3]. A decade later, Jaffe and Lichtenstein renamed it as chondroblastoma and clearly separated it from giant cell tumor [4].

Chondroblastoma can affect people of all ages. It is, however, most common in children and young adults between the ages of 10 and 20 years [5]. It is also more common in males than females [5]. Patients usually present with pain and swelling, particularly if a pathological fracture is present [5]. Association of chondroblastoma with aneurysmal bone cyst is well documented however in the cuboid this association has only once been reported. We report the case of a young adult who presented with a painful foot due to chondroblastoma associated with an aneurismal bone cyst.

## Case presentation

An 18 year old female presented to the clinic with a 4 month history of pain and swelling in the right foot. The patient had no history of trauma and denied any recent history of fevers and chills. Pain was insidious in onset and gradually worsened causing difficulty in walking. Pain had increased markedly during the two months prior to presentation to our clinic. The patient had a liver abscess at the age of 4 and past open heart surgery for total correction of tetralogy of fallot at the age of 7 years. On examination the patient had tenderness and a swelling on the outer aspect of the right foot in the calcanocuboid region. There was no soft tissue involvement even though the overlying skin was warm. No appreciable lymphadenopathy was noted. She could not bear weight on the right foot. Motor function was difficult to asses due to excruciating pain on movement; she would drag her foot on the ground while walking suggesting that dorsiflexion was severely affected.

**Figure 1:**
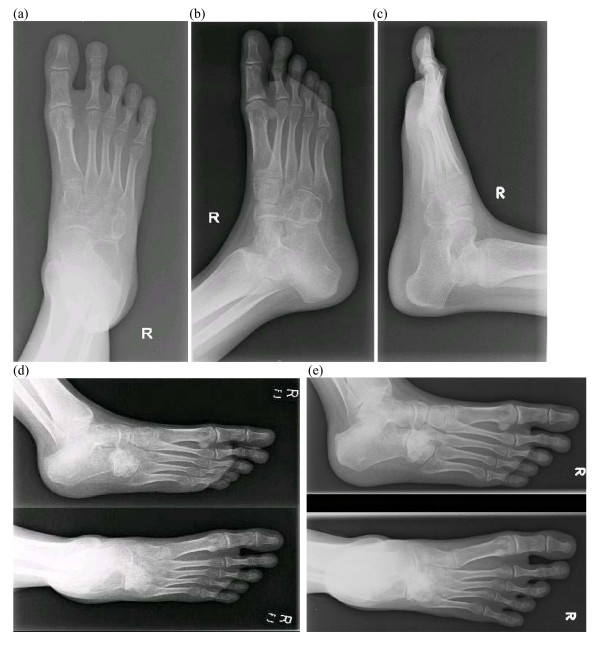
Conventional radiographs showed a lytic lucent area within the right cuboid bone. Septations were seen, but the cortex was intact. No fractures or dislocation of the cuboid was noted.

Conventional radiographs showed a lytic lucent area within the right cuboid bone (Figure 1a, 1b &1c). Septations were seen, but the cortex was intact. No fractures or dislocation of the cuboid was noted.

**Figure 2:**
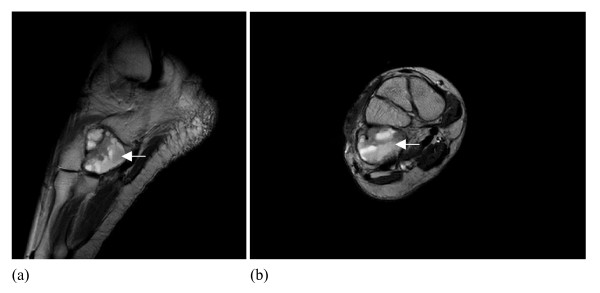
MRI showed cystic areas with fluid filled levels in the right cuboid bone. Mild expansion of the bone was noted. However no cortical break or involvement of other tarsal bones was seen.

MRI showed cystic areas with fluid filled levels in the right cuboid bone (Figure 2). Mild expansion of the bone was noted. However no cortical break or involvement of other tarsal bones was seen. The lesion appeared hypointense on T1 and hyperintense on T2 images and also showed post contrast enhancement. Based on the imaging studies the foremost differential diagnosis of giant cell tumor, chondroblastoma, and aneurysmal bone cyst were made.

**Figure 3:**
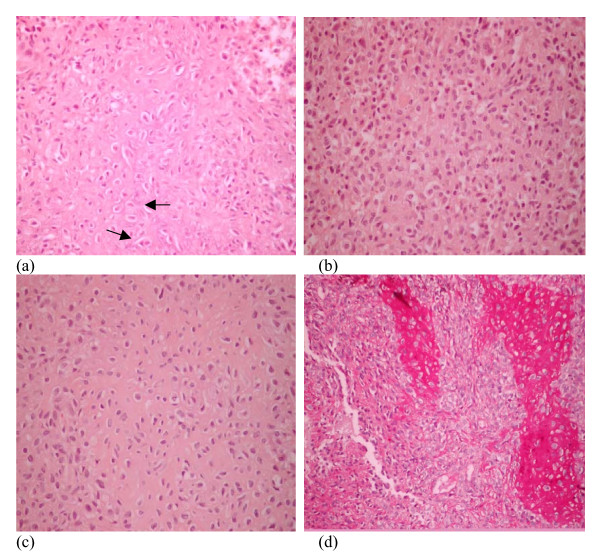
Microscopic examination of the tissue revealed sheets of cells exhibiting oval to elongated nuclei and moderate eosinophilic cytoplasm with distinct highlighted cell boundaries. Cells were indented with lobulated nuclei. Occasional mitotic figures and scattered giant cells were also identified. Focal areas of fine calcification around individual cells (called chicken wire calcification) were seen. Tumor cells were positive for glycogen on PAS stain. Cells were set in chondroid matrix which was highlighted with PAS stain.

An open biopsy was thereafter performed. Microscopic examination of the tissue revealed sheets of cells exhibiting oval to elongated nuclei and moderate eosinophilic cytoplasm with distinct highlighted cell boundaries. Cells were indented with lobulated nuclei (Figure 3). Occasional mitotic figures and scattered giant cells were also identified. Focal areas of fine calcification around individual cells (called chicken wire calcification) were seen (Figure 3a). Tumor cells were positive for glycogen on PAS stain. Cells were set in chondroid matrix which was highlighted with PAS stain (Figure 3). In some areas cystic spaces containing hemorrhage along with multinucleated giant cells in the wall were also seen. A diagnosis of chondroblastoma associated with an aneurysmal bone cyst was made.

An intralesional curettage using a high speed burr was then performed. The defect was then filled in by cancellous bone graft and bone substitutes.

At 6 months post-operatively the patient was ambulating normally without a limp. Range of motion at the ankle, subtalar, and midtarsal joints had improved significantly. The patient will be followed with serial radiographs at 3-months for 1 year. Thereafter, radiographs will be obtained every 6 months and then yearly until 5 years post-operatively. Her recent radiograph shows good incorporation of the bone graft.

## Discussion

Chondroblastoma is a benign tumor most commonly seen in the long bones specifically humerus, tibia, femur. Of all the bone tumors, chondroblastoma represents less than 1% with only 20% of it occurring in the talus or calcaneum [1]. Occurrence of an associated aneurysmal bone cyst has been reported to be as high as 33% [1]. Occasionally tumor may show a more aggressive pattern, with invasion of the joint spaces, adjacent bones, and very rarely, metastases [2] but generally it is a benign tumor. In 1931 Codman described 9 cases of epiphyseal giant cell tumor [3]. More than a decade later Jaff and Li Chenstein's research publication [4] made possible the acceptance of chondroblastoma as a separate entity and not a variant of giant cell tumor.

Chondroblastoma has been seen in people of all age groups. However, the pediatric age group and adults in their second decade of life appear to have a higher prevalence of the tumor [5]. It has been reported that a majority of the patients, i.e. 60% to 75%, are in their second decade of life [5].

Fink reviewed 322 cases of chondroblastoma in 1997 and found that only 42 involved the foot [6]. He also noted that in the foot chondroblastoma was more common in the posterior subchondral areas of the talus and calcaneus, as well as in the calcaneal apophysis [6]. The reason for this observation is not known.

Patients often present with insidious onset of pain. The site of the lesion not only determines effect of the tumor on the functionality of a patient but also the recurrence rate [7].

Typically on a radiograph a chondroblastoma presents with an eccentrically or centrally located osteolytic lesion that involves the epiphysis or other secondary ossification centers [8]. In 20% to 25 % of the cases metaphyseal involvement is also seen [7]. Cortical expansion, with erosion and periosteal reaction may be present and occasionally unusual radiological changes are also seen [9].

The tumor is composed of cellular and matrix rich areas. Chondroblasts which are round or polygonal cells with an oval or round nucleus and eosinophilic cytoplasm, make up the cellular areas. The nuclei are often indented and lobulated. In non-decalcified sections the chondroblasts appear focally delimited by a thin calcification rim, so called chicken wire [8]. Mitosis is always typical and is quite frequent in the cellular areas. Matrix rich areas are composed of different types of matrix: chondroid, osteoid, fibrous and rarely mature hyaline cartilage.

Treatment of chondroblastoma depends upon the anatomic location of the lesion and the extent of bone and/or joint involvement. It is usually treated by curettage and bone graft [5]. This procedure is curative in 90% of the cases. Other methods such as curettage alone, endoscopic curettage, endoscopic curettage with cementation, curettage with fat implantation, resection with allograft replacement, marginal resection radio-frequency ablation and osteochondral autograft transfer have also been used with some success [10]. Although the recurrence rate is reported to be 10 % to 15 % [5], this figure dramatically increases when accompanied by an aneurismal bone cyst, approaching almost 100% [11]. Open growth plates have also been considered as a risk factor for recurrence [10,12]. Some investigators have suggested that recurrence is secondary to less aggressive surgical curettage due to fear of injury of the physis [12] while other argue that recurrence might be related to anatomic location of the tumor rather than the method of the treatment [7]. A.J. Ramapa et al reviewed seventy-three cases of chondroblastoma treated between 1977 and 1998 and concluded that one possible explanation of recurrence of chondroblastoma in their case might be their anatomic location. Four out of eight tumors that recurred were located in the proximal femur and greater trochanter. The reason for this phenomenon could be difficulty in gaining access to these lesions due to their anatomy and a risk of damage of the blood supply to the femoral head [7]. However, the proximal femur and greater trochanter has been associated with lesions which are more aggressive, particularly cartilage tumors [10,13]. Previously chondroblastoma of the cuboid has been reported twice in the literature [14,15]. Sessions et al used both CT and MRI along with plain radiography and histopathology of the lesion for assessment. Their treatment of choice was curettage with a high speed burr followed by phenolization and cementation.

In our case we did an open biopsy followed by curettage, use of a high speed burr and bone grafting. Bone was harvested from iliac crest and was mixed with bone substitute to fill the cavity. No adjuvant therapy was done because the management of chondroblastoma is strictly surgical [16]. Although cementation provides better radiological follow up and has a low cost [15], we believe that biological reconstruction with autogenous bone graft is a superior option and has even better results as can be seen in our case and also supported by literature [17]. Although harvesting bone from iliac crest is associated with significant morbidity if adequate preoperative planning and a proper surgical technique are not implied [18]. Minor complications include superficial infection and minor hematomas at the wound site while in rare cases the procedure can lead to development of hernia, vascular injuries, nerve damage and deep infection [18].

## Conclusion

Chondroblastoma of the cuboid is a very rare and benign tumor, however, it should still be considered among the differential diagnosis whenever a neoplastic cause is being considered as the underlying pathology. Imaging techniques such as MRI scans and plain radiographs should be supplemented with an open biopsy for the final diagnosis. Managing chondroblastoma of the cuboid with an aneurysmal bone cyst with curettage, use of high speed burr and bone grafting has shown good outcomes. We believe that the risk of recurrence is best reduced by focusing more on doing an extensive curettage using the high speed burr rather than focusing on adjuvant therapy.

## Competing interests

The author(s) declare that they have no competing interests.

## Authors' contributions

YJS conceived of the case, drafted the manuscript and did the literature review. MU helped in drafting and reviewed the case. KM reviewed the case and helped in drafting the report. KH helped in literature review. All authors read and approved the final manuscript.

## Consent

The authors confirm that a formal written consent was taken for the publication of this case report.
